# Bioinformatic identification of lentivirus transfer plasmid contamination causing false-positive HIV NAT results in a high-throughput molecular screening laboratory

**DOI:** 10.1128/spectrum.02500-25

**Published:** 2026-02-20

**Authors:** Shannah Secret, Richard Mayne, Kaitlin Reid, Lilian Hook, Paul Lloyd-Evans, Victoria Maddox, Gail Miflin, Su Brailsford, Joanne Sell, Tanya Golubchik, Judith Breuer, Peter Simmonds, Heli Harvala

**Affiliations:** 1 Nuffield Department of Medicine, University of Oxford 105596, Oxford, United Kingdom; 2 Microbiology Services, NHS Blood and Transplant 9936, London, United Kingdom; 3 Cell, Apheresis and Gene Therapies, NHS Blood and Transplant 9936, Filton, United Kingdom; 4 Clinical Biotechnology Centre, NHS Blood and Transplant 9936, Filton, United Kingdom; 5 Technical and Scientific Development, NHS Blood and Transplant 9936, Filton, United Kingdom; 6 Sydney Infectious Diseases Institute, Faculty of Medicine and Health, University of Sydney 522555, Sydney, New South Wales, Australia; 7 Institute of Child Health, University College London 11700, London, United Kingdom; 8 Institute of Biomedicine, Medical Faculty, University of Turku and Turku University Hospital 169300, Turku, Finland; 9 Radcliff Department of Medicine, University of Oxford 371148, Oxford, United Kingdom; Hôpital Saint-Louis, Paris, France

**Keywords:** cross-reactivity, lentiviral vector, contamination, HIV, HIV nucleic acid testing

## Abstract

Blood screening programs require ultra-sensitive high-throughput PCRs to ensure the absence of pathogens to keep blood components safe for recipients. We present here details of an investigation wherein samples undergoing screening using a high-throughput multi-pathogen NAT assay began to exhibit an abnormally high HIV false reactivity rate. NHS Blood and Transplant usually sees up to 10 HIV reactive samples per year, but this increased to over 100 in just a few days. Investigations into the cause were imperative to prevent recurrence. Analyzer and reagent issues were ruled out and environmental swabbing revealed extensive contamination of the laboratory, with 356 of 392 swabs testing positive. We demonstrate the methods by which we used amplicon NGS and a custom bioinformatic approach to differentiate between expected amplicons, PCR artifact, and contaminant sequences from extracted false-positive samples. Our investigation was able to trace the source of contamination to an unexpected source, a lentivirus transfer plasmid, containing the long terminal repeat (LTR) region of HIV-1, from a neighboring laboratory. This incident demonstrates the risks of false reactivity from HIV-derived lentiviral vectors, which has also been seen in patients receiving lentiviral vectors as part of gene or CAR T-cell therapies. The nature of the contaminant meant that there was no risk to donors, recipients, or staff. It did, however, demonstrate the critical importance of facility design and operation in plasmid manufacturing sites to prevent the spread of such contaminants and avoid unexpected downstream consequences such as those encountered in the screening laboratory.

**IMPORTANCE:**

This paper describes a large-scale contamination incident that occurred at a critically important high-throughput screening laboratory resulting in a significant spike of positives in the HIV screening. We describe the use of NGS and a custom bioinformatics approach that enabled us to identify the unexpected source of the contamination—a lentivirus transfer plasmid, containing the long terminal repeat (LTR) region of HIV-1, being produced in a neighboring laboratory. This incident demonstrates the risks of false reactivity from HIV-derived lentiviral vectors, which has also been seen in patients receiving lentiviral vectors as part of gene or CAR T-cell therapies. The nature of the contaminant meant that there was no risk to donors, recipients, or staff. It did, however, demonstrate the critical importance of facility design and operation in plasmid manufacturing sites to prevent the spread of such contaminants and avoid unexpected downstream consequences such as those encountered in the screening laboratory.

## INTRODUCTION

High-throughput screening plays an increasing role in the diagnosis of infectious diseases, ensuring blood safety, and monitoring public health. In blood donation, donor selection and testing are used as part of the risk reduction strategy. Both high-throughput nucleic acid testing (NAT) and serological screening are used for identifying pathogens and preventing transfusion-transmitted infections (1). In England, NHS Blood and Transplant (NHSBT) screens over a million blood donations each year. This includes NAT for HIV-1 and HIV-2 RNA, HCV RNA, and HBV DNA, currently carried out on the Roche Cobas MPX assay on the Cobas 6800/8800 platform. In a typical year at NHSBT, on average, 10 samples are HIV RNA reactive on this assay. Of these, 5–10 are then confirmed through additional testing as a true HIV infection in a donor. The remaining reactive samples that are not confirmed as true positives are considered false reactives (FR). The reason for the false reactivity is unknown and not investigated further, since all HIV-reactive donations, whether confirmed or not, are removed from the blood supply.

The large-scale nature of high-throughput NAT presents challenges, including false positives and cross-contamination. Contamination can be highly persistent and is easily dispersed as airborne aerosols and through contact points, making it difficult to manage and eliminate once it has occurred (2, 3). Effective control typically requires well-considered workflows with separated space and work force, extensive decontamination with strong bleach solutions and ultraviolet light, and enhanced engineering controls to prevent recurrence (2, 4). Identifying the source of contamination is important for preventing recurrence but can be challenging, and typically it is extremely rarely identified, especially in complex research or diagnostic environments with multiple nearby laboratories producing and analyzing amplicons (5).

The vulnerability of such high-throughput screening at NHSBT was demonstrated in June 2023 and again in January 2024, when large peaks of suspected FRs occurred over very short time periods. This substantially interfered with testing workflows, causing a backlog of screening; some testing had to be stopped and diverted to another laboratory, while cleaning and investigations were conducted.

Here, we describe the investigation of this incident along with background data on blood donation screening for HIV by NHSBT between 2019 and 2024. We describe the use of novel next-generation sequencing (NGS) and bioinformatics analysis tools used to identify the contamination source.

## MATERIALS AND METHODS

### Blood donation screening for HIV

Plasma samples obtained from all blood donations in England are screened in pools of 24 donors using the Roche Cobas MPX test. It is a real-time multiplex PCR for HIV RNA (including HIV-1 group M and O, HIV-2), HCV RNA, and HBV DNA. It targets two different regions of the HIV-1 genome. Plasma samples are also screened simultaneously for HIV Ag/Ab, and any sample with detectable HIV RNA or antibodies is sent for further confirmatory testing with alternative NAT and serology tests at the NHSBT Microbiology Services Laboratory. If HIV infection is confirmed, the donor is contacted and referred for specialist care. In cases where HIV RNA is only detected at screening but not at the reference laboratory, and all serological testing remains also negative, any potential risk factors for HIV infection will be obtained from donor and repeat samples requested for further testing, including HIV proviral DNA. In cases where all molecular and serological testing is negative in the repeat sample, this initial HIV RNA positivity in the index sample will be considered false. Blood donation data on initial HIV RNA reactives and confirmed HIV infections are collated and monitored by the NHSBT Microbiology Surveillance team. We report relevant data from January 2019 to January 2024.

### Further testing of initial HIV RNA-positive samples shown to be false reactives

PCR products from the Cobas MPX assay, including three FR samples and each of the three run controls, were run on agarose gel electrophoresis to visualize the number and size of PCR products. The three run controls consisted of PCR product MPX control (HIV-1 M-group control, HBV, and HCV), HIV-1 O-group control, and the HIV-2 control. The PCR products were then subjected to sequencing.

### Extraction of environmental swabs

A total of 392 environmental swabs were taken from a full range of sites in the affected laboratory, including lab benches, laboratory coats, door handles, testing equipment, cleaning and maintenance equipment, and consumables. These swabs, together with 66 clean swabs, were tested by Roche in Switzerland. The swabs had 1.6 mL Roche Specimen Diluent added and were incubated at RT overnight. One milliliter was then tested with Cobas MPX. PCR plates were subsequently sent to the University of Oxford for direct sequencing, whereas any remaining material was forwarded for extraction using Zymo quick viral DNA/RNA extraction kit (R1041, Zymo Research) and eluted in 20 µL nuclease-free water. Extracts were stored at −70°C until use.

### Sequencing and bioinformatic analysis

PCR products from seven blood donor samples found false reactive for HIV RNA by Cobas MPX assay, and from four environmental samples found to be reactive similarly in Cobas MPX, were selected for sequencing. PCR products were selected across different assay plates, with ct values varying from 33.9 to 39.8. In addition, all three controls from the multi-pathogen NAT assay (multi-pathogen control for HIV-1 M-group, HBV, HCV; HIV-1 O-group; HIV-2) and two negative controls were included. PCR products were quantified using the Qubit (ThermoFisher Scientific) and diluted to 20 ng/µL. These were then sent to GENEWIZ by Azenta Life Sciences (Germany) for Amplicon-EZ NGS on an Illumina MiSeq platform using a PE250 strategy at a maximum read depth of 5 × 10^4^ per sample.

Sequencing data were analyzed using Castanet version 8.3 (6) with the modified amplicon pipeline. No pre-filter was used, and trim settings were set through the estimation of fragment size, based on QC results provided by the sequencing facility. The mapping reference provided included a range of representative reference viral genomes, including for prevalent genotypes of HIV-1, HIV-2, HBV, and HCV. Mapped reads were cleaned by excluding unaligned portions of read fragments (soft clips), mapped fragments excluding soft clips, and mapped fragments where base identity with reference sequences did not exceed 60 bases; this value was chosen because it is more than double the anticipated length of a primer sequence. Non-viral origin sequences were discarded.

Selection of optimal bioinformatics parameters to identify LTR reads was performed by iteratively reducing the mapped fragment length and observing the pipeline’s alignment plots. This refinement process revealed that contaminant LTR reads frequently formed chimeras with other, longer amplicons (predominantly PCR products) in the sample and were consequently filtered from results using default parameters. Castanet amplicon pipeline settings were set as default except for the following: fragment minimum length = 36, Q score cut-off = <20, adapter mismatch = 2 bases, palindromic alignment mismatch = 10 bases, non-paired read alignment mismatch = 7 bases, and filter soft clips = True. In addition to allowing users the ability to filter amplicons by mapped length, the Castanet amplicon pipeline allows users to filter soft-clipped read fragments by parsing each mapped read’s compact idiosyncratic gapped alignment report (CIGAR string). As the software’s underlying mapping software (BWA-mem2) (7) allows for multiple primary alignments per read, its use in combination with soft-clip removal enables precise characterization of distinct fragments in chimeric reads, provided appropriate mapping references are supplied for each component.

### Plasmid-specific PCR of environmental swabs

A real-time PCR was designed targeting two different regions of the plasmid suspected to be the contaminant (primer sequences and PCR cycle details not shown for commercial reasons). Extracts from the environmental swabs were then tested with the plasmid-specific PCRs. SYBR Green PCR Master Mix (SYBR Green Universal Master Mix, Applied Biosystems) was used with a total reaction volume of 20 µL, including 2 µL of template and primers at a final concentration of 0.5 µM. Real-time PCRs were conducted on a Quantstudio 1 (ThermoFisher Scientific) on 11 environmental swabs. The positive PCR amplicons then underwent Sanger sequencing for comparison to the suspected contaminant.

## RESULTS

### The contamination incident with background data

**Fig 1:**
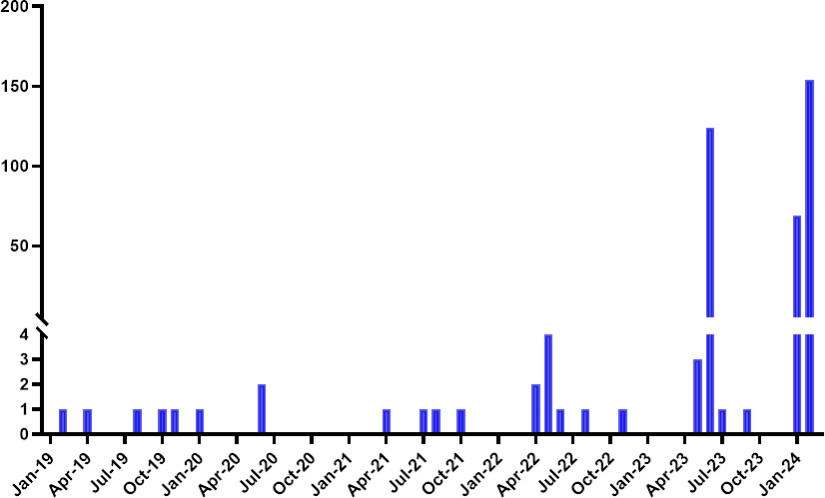
The number of false reactive HIV NAT tests during blood screening from 2019 to 2024.

Between January 2019 and January 2024, a total of 35 confirmed HIV-positive donors were identified by NHSBT (9 in 2019, 9 in 2020, 9 in 2021, 8 in 2022, 5 in 2023, and 0 in January 2024). In the same period, 369 FRs were identified (5 in 2019, 3 in 2020, 4 in 2021, 8 in 2022, 126 in 2023, and 223 in January 2024) (Fig. 1). From these, 124 FRs were observed over a period of 8 days in summer 2023, and another peak of 223 FRs was observed within a one-month period in January 2024. These episodes led to 347 blood donations being discarded.

### Gel electrophoresis analysis of assay products

**Fig 2:**
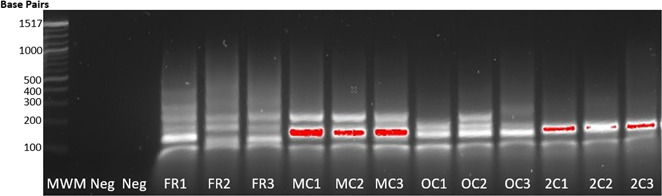
2% agarose gel showing bands from three false-positive samples in comparison with control samples from NAT assay. MWM: molecular weight marker (Quick-Load Purple 100 bp DNA ladder, New England Biolabs); Neg: negative control (buffer); FRx: false reactive samples; MCx: MPX control containing HIV-1 M-group control; OCx: HIV-1 O-group control; 2Cx: HIV-2 control.

Amplicons from three FRs, three MPX controls (including HIV-1 M-group control), three HIV-1 O-group controls, and three HIV-2 controls were analyzed to determine whether, and how much, amplicon DNA was present (Fig. 2). The gel image demonstrates that there was clearly a substantial amount of amplified DNA in each sample. The Cobas MPX control should contain three or more amplicons, and this was consistent with the observation of multiple bands in the range of 100–200 bps. However, multiple bands were also observed in the three FR samples, inconsistent with the expected one or two bands if they were positive for HIV-1 or HIV-2. Further analysis of FR samples and PCR-negative samples by gel electrophoresis was inconclusive, with amplicon bands frequently found in PCR-negative samples (data not shown), suggesting that a large proportion of the observed low-sized DNA was non-specific products.

### Environmental swabbing shows the extent of contamination

Three hundred fifty-six of 392 environmental swabs were reactive in the HIV component of the Cobas MPX assay (ct values: 20.91–42.76, mean 32.04). None of the swabs taken were reactive for HBV or HCV. In addition, 66 unused swabs were all negative for all targets.

### Short read NGS sequencing of products

**Fig 3:**
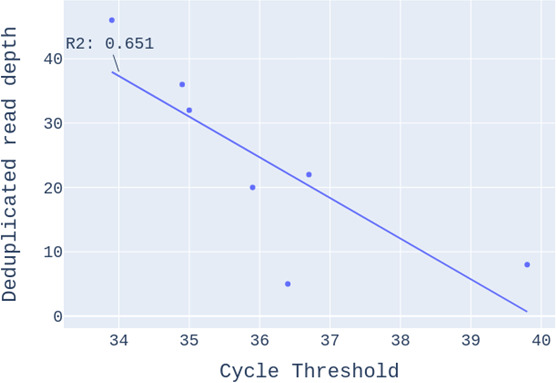
Read count of false reactive samples against cycle threshold (ct) values (*N* = 7).

A selection of samples, consisting of four environmental swabs, seven FR samples, two negative controls, and three positive controls, was analyzed by Illumina (short-read) NGS on the MiSeq platform using 2 × 250 paired end. Counts of reads mapped to HIV-1 were low compared to the total sequencing depth (Table 1). Read depths were nonetheless sufficient to support the analyses. In addition, the read numbers were correlated with the ct value (Fig. 3).

**TABLE 1:** Ct values and respective read totals for PCR amplicons of false reactive (FR) samples, environmental swabs (ES), and positive controls provided for the investigation

Sample	Roche Cobas MPX Ct value	Read totals in Roche PCR products			
		HIV-1 gag	HIV LTR	HBV	HCV
Controls					
HIV-MPX control		136	98	4,125	454
HIV-O control		44	0	0	0
HIV-2 control		0	0	0	0
FR1	33.9	0	46	0	0
FR2	34.9	0	36	0	0
FR3	35.0	0	32	0	0
FR4	39.8	0	8	0	0
FR5	36.7	0	22	0	0
FR6	35.9	0	20	0	0
FR7	36.4	0	5	0	0
Neg1	>40	0	0	0	0
Neg2	>40	0	0	0	0
ES1	23.5	0	352	0	0
ES2	25.5	0	359	0	0
ES3	23.0	236	399	0	0
ES4	25.4	0	531	0	0

As expected, large numbers of HIV, HCV, and HBV reads were observed in the HIV-1 M-group multiplexed positive control sample (Table 1). A similarly substantial number of HIV-1 O-group reads were obtained in the LTR region from the HIV O-group control. However, no reads identifiable as HIV-2 LTR or elsewhere in the genome were observed in the reads returned for the HIV-2 positive control. Reads could be readily assembled to create a uniform length consensus sequence in two regions of the HIV-1 genome (lLTR and *gag* regions) from the multiplex control, along with the 5′UTR of HCV and the *pol* gene of HBV (Table 1). Similarly, an HIV-1 O-group LTR sequence could be assembled from the corresponding positive control. Inferred amplicon lengths and similarity to the closest published sequences available on GenBank are shown in Table 2. While both *gag* and LTR sequences were amplified and detected by NGS in the amplicons from the Roche Cobas MPX positive control, only LTR sequences were amplified from the FR and from environmental samples. From this, it was suspected that the LTR sequences in the FR and environmental samples arose from contamination with an HIV-1 LTR-containing clone or a specific LTR amplicon, rather than from an HIV-1 virus contamination source.

**TABLE 2:** Mapping of consensus sequences to HIV-1, HBV, and HCV reference sequences

Sample	Virus	Reference^*a*^	Type^*b*^	Region	Length	Identity
Roche MPX control (A1)	HIV-1	KM248765	CRF73_BG	LTR	134	98.0%
		AF033819	B	*gag*	156	100%
	HCV	AF009606	1a	5′UTR	241	100%
	HBV	AF143304	A2	*pol*	131	100%
O group	HIV-1	MH705150	O	LTR	143	99.5%
FR1	HIV-1	AF033819	B	LTR	134	100%
FR2		AF033819	B	LTR	134	100%
FR3		AF033819	B	LTR	134	100%
FR5		AF033819	B	LTR	134	100%
FR6		AF033819	B	LTR	134	100%
FR7		AF033819	B	LTR	134	100%
ES1		AF033819	B	LTR	134	100%
ES2		AF033819	B	LTR	134	100%
ES3		AF033819	B	LTR	134	100%
ES4		AF033819	B	LTR	134	100%

^*a*^ Accession numbers of closest matching sequence on GenBank.

^*b*^ Subtype (HIV-1, HCV) or genotype (HBV).

The LTR amplicon sequence of the Roche Cobas MPX control was distinct from corresponding sequences from the FR by over 20 nucleotides (total length 134 nucleotides; data not shown for commercial reasons). Its sequence was most closely similar to a complex recombinant form (CRF73_BG), while the sequence amplified in the *gag* region was identical to the subtype B reference sequence, AF033819. The HIV-1 O-group sequence was most closely related (one base difference) to the HIV-1 O isolate 20-02 from Cameroon, a region of Africa where most HIV-1 O-group isolates have been obtained to date. Notably, LTR sequences in both Roche controls appear to have been amplified by an alternative sense primer matching the HIV-1 O-group sequence rather than the primer matching the HIV-1 IIIB strain (previously known as HTLV-IIIB), reference sequence AF033819. This was extended by four bases at the 5′ end and contained four sequence differences from the M-group reference.

The seven FR samples all returned reads in the HIV-1 LTR (Table 1). A substantial proportion of reads were incomplete, and there was a much higher frequency of misincorporated bases (read error) in the sequences than normally observed in Illumina-derived sequences, as would be expected when sequencing very short fragments. The reads from FR4 were particularly poor in quality and were therefore omitted from further analysis. For the remainder, assembled sequences spanned the same regions in the LTR as the M group positive control, although some amplicons incorporated the HIV-O group alternative sense primer. Sequences within the amplicon were all identical to the HIV-1 IIIB reference sequence, AF033819.

### Sequence comparison of potential contaminants

It was then investigated whether any of the three lentivirus transfer plasmids being produced in high quantities in a neighboring laboratory was the source of the contamination. LTR and *gag* sequences from the FR and environmental samples were compared to those from the three lentivirus transfer plasmids. Comparison revealed that plasmid 1 had an identical LTR sequence to the HIV-1 reference strain, AF033819, and to the six consensus sequences derived from the FR samples, while the others all showed a single-base difference in the LTR sequence. Plasmid 3 contained an HIV-1 *gag-*encoding sequence (but no LTR); however, the sequence in the *gag* amplicon region was distinct from the HTLV-IIIB clone sequence, the Roche Cobas MPX positive control, and environmental sample 3.

### PCR confirmation of presence of lentivirus transfer plasmid in environmental swabs

All of the 11 environmental swabs tested were positive in the plasmid-specific PCRs for at least one of the two regions tested (Table 3). All amplicons from these were sequenced and were identical to the corresponding regions in this lentivirus transfer plasmid.

**TABLE 3:** Environmental swab sample locations and HIV-1 PCR results, reported as NEG (negative) or with ct values for positive samples

Swabbing location	Roche Cobas MPX	Plasmid region 1	Plasmid region 2
In lab roller door cabinets: vacuum cleaner Henry	28.40	34.5	36.0
NAT working area: printer area	25.39	30.1	29.4
6800 top outside cover analytic module	25.8	29.8	NEG
NAT working area: draws below front bench (interior)	27.26	32.3	NEG
NAT working area: draws below front bench (interior)	29.9	NEG	29.2
In lab roller door cabinets: vacuum cleaner Henry	28.40	34.5	36.0
Waste room: floor near fan	22.45	29.4	28.3
Waste room: fan	25.0	32.5	30.9
NAT working area: drawers below standalone data manager	31.93	NEG	34.7
NAT working area: handle of NAT reactive material freezer	31.84	36.2	32.7
NAT working area: drawer handles below pre-working area	29.51	33.7	31.8
Positive PCR plate (Roche)		NEG	NEG
Negative extraction control		NEG	NEG
Negative extraction control		NEG	NEG

## DISCUSSION

The repeated occurrence of suspected FR samples tested using the Roche Cobas MPX assay led to severe problems with testing workflows in June 2023 and January 2024. The Cobas 6800/8800 platforms were thoroughly inspected and found to be working as expected, indicating the likelihood that the FRs arose from sample or external contamination, as opposed to a machine or assay issue. Standard investigations were performed to identify what was suspected to be HIV-1 contamination at the time.

Several hypotheses for the source of the contamination were proposed. They included positive controls, external quality assurance (EQA) samples, a high-titer HIV-positive donation sample, amplicons from previous runs, or malicious interference. Neither a high titer positive nor a positive EQA sample had been encountered in the laboratory in the time leading up to the incident. Amplicon carryover was also deemed unlikely, since the Roche Cobas MPX assay uses dUTP instead of dTTP during amplification, allowing uracil-DNA glycosylase (UDG) to digest any contaminating amplicons prior to amplification. Amplicon carryover is assumed to be largely eliminated by this process, but to what extent is unclear.

An insight into the source of external contamination was provided by genetic comparison of amplicon sequences with those of its suspected sources. A substantial problem was the lack of information on the primers and genomic regions of HIV-1 and other multiplexed targets amplified by the Cobas MPX assay. It was known, however, that the assay targets two regions of the HIV genome across different groups but uses the same fluorophores for all targets. It was therefore unknown *a priori* whether the contaminant in FR samples was HIV-1 M- or O-group or even potentially HIV-2; further, it was also unclear if HIV false-positivity arose from contamination of PCRs for one or both target regions.

Without knowledge of the nature of the amplicons, we decided to adopt a NGS approach, where amplicons were sequenced using metagenomic approaches by Illumina. In this method, PCR product can be amplified and sequenced without prior knowledge of the target sequences. Returned paired-end reads can therefore be interrogated by bioinformatics pipelines to identify the reads against human and virus databases and to segregate them for further analysis. With this, we demonstrated that amplicon LTR sequences obtained from the seven FR and four environmental samples were identical to the HIV-1 strain IIIB reference sequence, AF033819, and distinct from the Roche Cobas MPX control amplicon. The AF033819 sequence derives from the original HTLV-III isolate of HIV-1 reported when HIV-1 was first characterized by Montagnier and colleagues (8). Given the rapid evolution and substantial accumulated sequence drift of HIV-1 over the following 40 years, a sequence identical to HTLV-III could not possibly be circulating in the UK and elsewhere. Furthermore, whereas both *gag* and LTR sequences were amplified from the Roche Cobas MPX positive control (as expected), only LTR sequences were amplified from the FR samples and all except one environmental sample. The *gag* PCR yielded more reads than the LTR in the positive control (Table 2), which indicated that this was not a differential sensitivity issue between the two regions.

These findings were consistent with a laboratory, rather than an exogenous, source of contamination, such as originating from sample or amplicon carryover from an HIV RNA-positive sample. Plasmid contamination was suspected since lentiviral vectors containing retroviral LTRs are typically derived from the prototype HTLV-IIIB clone sequence, and this was confirmed by sequence comparison between lentivirus transfer plasmids and the FR samples. Furthermore, the same contamination was detected in all the environmental swabs tested with a plasmid-specific PCR.

To date, we are not aware of any other reports of HIV-1 sequence containing lentivirus transfer plasmids causing contamination of a testing facility. There are, however, several published case reports of false-positive HIV reactivity in patients receiving lentiviral vector-based gene and CAR T-cell therapies (9, 10). HIV NAT assays commonly target conserved regions in the LTR and *gag* regions, also found in lentiviral vectors, resulting in false positivity across NAT assays from several manufacturers (11). Due to the proprietary nature of both HIV NAT assays and commercially manufactured plasmids, it is often not possible to obtain the sequences to predict cross-reactivity. It was only due to the excellent cooperation of the NAT assay and plasmid manufacturers that we were able to resolve this incident.

Due to the nature of this incident, a rapid clinical risk assessment was carried out. There was no risk to blood component recipients at any time during this incident, as all donations identified as suspected FR were discarded. To avoid missing a true HIV-positive donation during this time, the usual confirmatory testing was conducted. All FR samples were negative for HIV serology at screening and in further testing, including an alternative NAT and two serological assays, demonstrating that no true HIV-positive donations occurred during this time. Donors were deferred until all these results had been reported. The demonstration, by the results of the environmental swabbing, that the contamination was extensive and widespread also raised concerns about any risk to staff or visitors working in the contaminated laboratory. However, the HIV-1 LTR element within the transfer plasmids, identified as the likely source of this contamination incident, is not harmful and cannot cause infection in those exposed to it via any possible route. Reassurance to staff and enhanced cleaning using 0.5% bleach, known to destroy plasmids, were implemented.

Investigations into how the lentivirus transfer plasmid contamination entered the testing laboratory were not able to identify a specific single cause. However, it is important to acknowledge that plasmids were manufactured in an industrial scale in a facility adjacent to the screening laboratory. Although no laboratory or other staff is shared between these facilities, the laboratories are interconnected by a corridor which was also noted to be contaminated. Furthermore, the route to the shared canteen from the manufacturing facility is also adjacent to the microbiological screening laboratory, highlighting the possibility that the contamination may well have been transmitted by laboratory workers with access to both laboratories. This was further evidenced by finding that the screening laboratory, laboratory coats, and lockers were all found to be heavily contaminated. It is also likely that the lentivirus transfer plasmid had been transmitted through the air, as contamination was detectable on overhead gantry, air-conditioning units, and throughout the floor area—all areas unlikely to be touched. The investigations into this are still ongoing and are likely more complicated than initially considered, noting that these facilities have different air handling units. Since this incident, several changes have been implemented to prevent a recurrence. These have included enhanced cleaning of the laboratory and the introduction of daily change of laboratory coats, in addition to workflow changes in which staff producing plasmids are no longer allowed to enter the screening laboratory. The manufacture of the lentivirus transfer plasmids has also been paused, while investigations continue into methods to mitigate and prevent future contamination.

This investigation demonstrated the need for non-conventional bioinformatic approaches. Amplicon short-read sequencing provided a rapid and effective method to investigate the source of contamination in the absence of any knowledge of the sequences of the primers used in the PCR for target amplification. However, it is commonplace to include at least one cleaning step in bioinformatic pipelines to remove “unwanted” sequence fragments, which may involve adapter trimming, e.g., Trimomatic (12), read labeling Kraken2 (13), or context-specific background reduction, such as Decontam (14). Although most such tools use far more parameters than just mapped fragment size to filter on, it is commonly recommended to remove fragments of length <70 nt from downstream analyses, as this size range corresponds to artifact such as primer dimers and contaminant airborne amplicons (15). Our contaminant LTR sequences were automatically removed by the standard Castanet pipeline parameters as a result of this conventional wisdom, highlighting how unconventional bioinformatics approaches and understanding are required when using NGS as a troubleshooting tool.

In conclusion, we have identified a highly unexpected source of contamination problem in a high-throughput and critical screening facility in NHSBT. The investigation highlights the fact that molecular laboratories are extremely vulnerable to plasmid contaminations and the need for careful design and physical separation of plasmid manufacturing facilities. It also underlines the role of laboratory reference facility testing expertise and the need for advanced molecular and bioinformatic skills to resolve potential contamination issues.

## Data Availability

Data are available at EBI ENA/NCBI/DDBJ under accession number PRJEB101388.
